# Trunk Stability, Trunk Strength and Sport Performance Level in Judo

**DOI:** 10.1371/journal.pone.0156267

**Published:** 2016-05-27

**Authors:** David Barbado, Alejandro Lopez-Valenciano, Casto Juan-Recio, Carlos Montero-Carretero, Jaap H. van Dieën, Francisco J. Vera-Garcia

**Affiliations:** 1 Sport Research Centre, Miguel Hernández University, Elche, Spain; 2 MOVE Research Institute Amsterdam, Department of Human Movement Sciences, Vrije Universiteit Amsterdam, Amsterdam, the Netherlands; University of Alabama at Birmingham, UNITED STATES

## Abstract

Although trunk muscle function has been suggested to be a determinant of judo performance, its contribution to high-level performance in this sport has been poorly studied. Therefore, several tests were used to assess the differences in trunk muscle function between 11 international and 14 national level judo practitioners (judokas). Trunk strength and endurance were assessed using *isokinetic tests* and core stability was assessed using two protocols: 1) *sudden loading*, to assess trunk responses to unexpected external perturbations; 2) *stable and unstable sitting*, to assess the participants’ ability to control trunk balance. No differences between groups were found for trunk flexor isokinetic strength, trunk responses against lateral and posterior loading and trunk control while sitting. However, international level judokas showed significantly higher trunk extensor isokinetic strength (p <0.05) and lower trunk angular displacement after anterior trunk loading (p <0.05) than national level judokas. Few and low (r < 0.512) significant correlations were found between strength, endurance and stability parameters, which suggests that trunk strength and endurance are not limiting factors for trunk stability in competitive judokas. These results support the importance of trunk extensor strength and trunk stability against forward perturbations in elite judo performance.

## Introduction

Competitive judo is a high-intensity sport in which the athletes are constantly pulling and pushing each other while performing different techniques (throws, pins, chokes, arm bars, etc.) [1–3]. It has been recognized that judo is a complex sport with demands comprising a number of specific characteristics to achieve a high level in competition.

Upper and lower body strength and endurance, speed, anaerobic power and trunk muscle function have been pointed out as important factors to be successful in judo competition [4, 5]. Regarding trunk muscle function, improving trunk strength and endurance would allow judo practitioners (judokas) to increase their ability to generate and maintain force throughout a fight. In addition, core stability might contribute to judo performance as it would facilitate the transmission of forces generated by the lower body to the upper body (and vice-versa) [6] during judo techniques and it would enhance balance control [7], a key factor in coping with disturbances caused by the adversary [1–3].

While plausible, the importance of trunk muscle function to judo performance remains to be proven. In previous studies on trunk muscle strength, international and national level judokas showed similar flexor and extensor isokinetic strength [4], while adult, amateur judokas showed higher hip extension and trunk flexion isometric strength than junior (sub-19 years) and cadet (sub-16 years) judokas [8]. In this last study, differences between groups could, however, be due to age. Moreover, no relation between trunk strength and performance level within groups were found in this study [8]. When judokas and athletes from other sports were compared, elite judokas showed a higher trunk isokinetic strength than cyclists [9]. Regarding trunk muscle endurance, judokas showed sit-up test scores [10–12] above the 80th percentile of normative data [13]. Finally, brown and green belt judokas showed higher whole-body stability against sudden posterior perturbations than recreational athletes [14], but no differences between both judo groups were found and trunk stability was not tested. To the best of our knowledge, no other studies comparing judokas of different performance levels (e.g., recreational vs. competitive level) have been done.

The aim of this study was to assess the relationship between judo skill level and trunk muscle function. Specifically, trunk isokinetic strength and endurance, trunk responses to unexpected external perturbations and trunk movement control were compared between national and international competitive level judokas. In addition, the relationships between trunk strength, endurance and stability parameters were examined to better understand trunk muscle function in competitive judokas.

## Methods

Twenty-five male judokas participated voluntarily in this study. All of them were black belt with more than 7 years of experience and a work out frequency of 3–5 days per week. The judokas were divided into two groups according to their level: 14 national level judokas (age = 24.0 ± 8.5 years; years of judo practice = 14.1 ± 5.6 years; mass = 74.4 ± 9.9 kg; height = 1.74 ± 0.07 m; trunk moment of inertia = 5.0 ± 0.8 kg*m^2^) and 11 international level judokas (age = 24.5 ± 6.1 years; years of judo practice = 13.7 ± 6.1 years; mass = 75.2 ± 13.1 k; height = 1.72 ± 0.07 m; trunk moment of inertia = 5.2 ± 1.2 kg*m^2^). International level judokas were those that had been selected by the Spanish national team to participate in international tournaments in the last 3 years, or had won a medal in a national tournament. All national level judokas had competed in national tournaments but had not won medals in these. Participants gave written informed consent to participate in this study and to include their images in the figures of this manuscript. The experimental procedures used in this study were in accordance with the Declaration of Helsinki and were approved by the University Office for Research Ethics.

**Fig 1:**
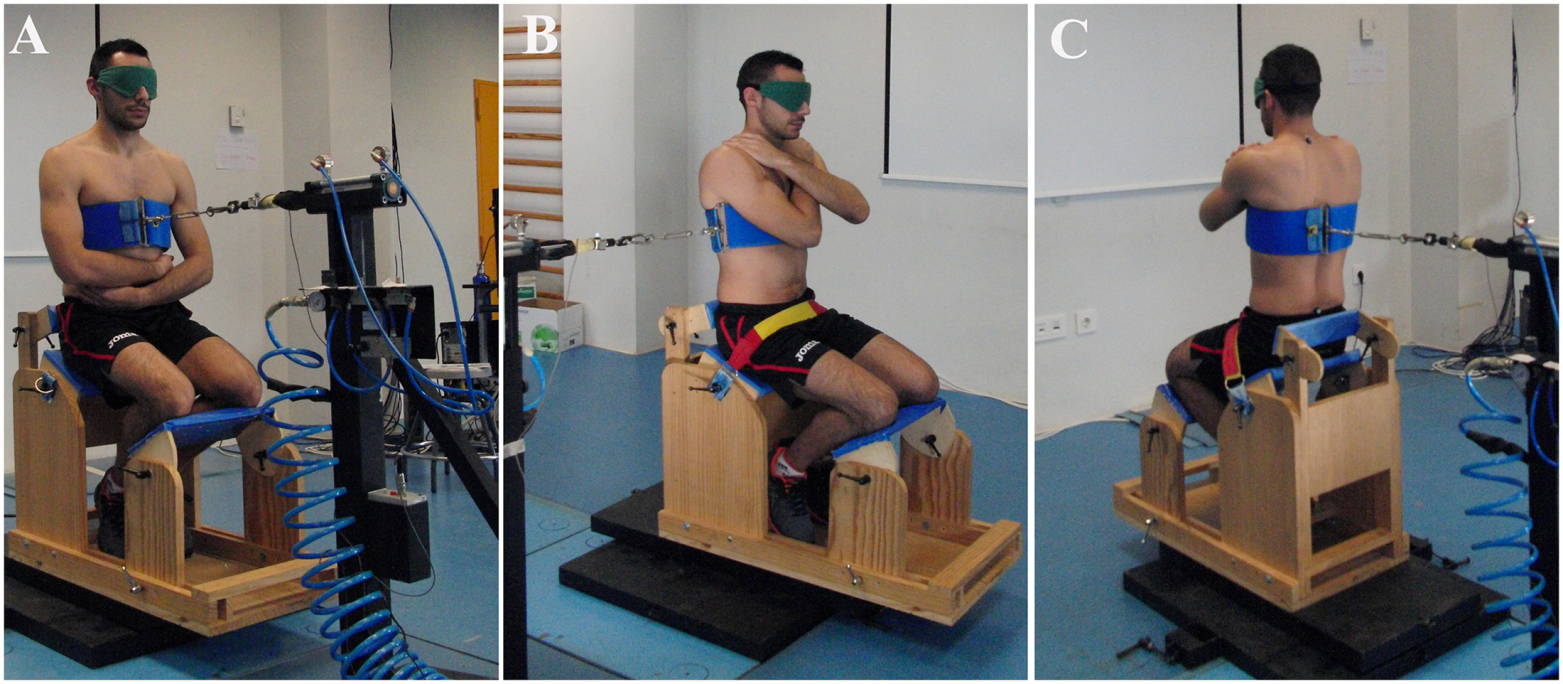
Set-up for applying loads using a pneumatic pulling mechanism in the 1A) posterior, 1B) anterior and 1C) lateral loading direction.

Several tests were used to assess trunk muscle performance. Two protocols were performed to evaluate different trunk stability parameters (in the following order): 1) *Sudden loading protocol* to assess trunk responses to unexpected external perturbations in anterior, posterior and lateral (right side) direction. Participants were placed in a seated position in a wooden chair that restricted hip motion while leaving the trunk free to move in all directions (Fig 1). A pneumatic piston, attached to a harness via a steel cable tensioner, pulled with 4.2 bars of pressure and 0.5 m/s of speed to load the trunk. The cable was aligned horizontally with the centre of mass of the upper body (referred to below as HAT) [15]. The magnitude and timing of the perturbation was measured using a load-cell (MLP-100, Transducer Techniques Inc., Temecula, CA, USA), attached to the piston and located in-series with the cable tensioner and the harness. The force signals were amplified, and A/D converted (16 bit resolution over ±5 V) at 1000 samples/s. Biofeedback of load-cell forces was provided to the researcher in real time to keep participant’s forces constant (25–27.5 N) prior to the sudden perturbation. Participants were instructed to maintain a neutral spine posture without pulling on the load-cell before sudden loading and not to respond voluntarily to the perturbation. Trunk kinematics were recorded at 200 samples/s with seven T10 cameras of the Vicon 3D-motion analysis system (Vicon MX, Oxford, UK). Three passive retro-reflective markers were attached at the following locations: one over the L5 spinous process, and two on the harness, approximately 2 and 4 cm to the right of the HAT centre of mass. Data were reconstructed using Nexus 1.8.2 software (Vicon MX. Oxford, UK). Five sudden perturbations were applied to the trunk in anterior, posterior and lateral direction, with one minute rest between trials and 5 minutes rest between directions. Each perturbation took place without warning within a 15 seconds window. Participants were blinded with a mask. The order of the perturbation directions was balanced over participants.

**Fig 2:**
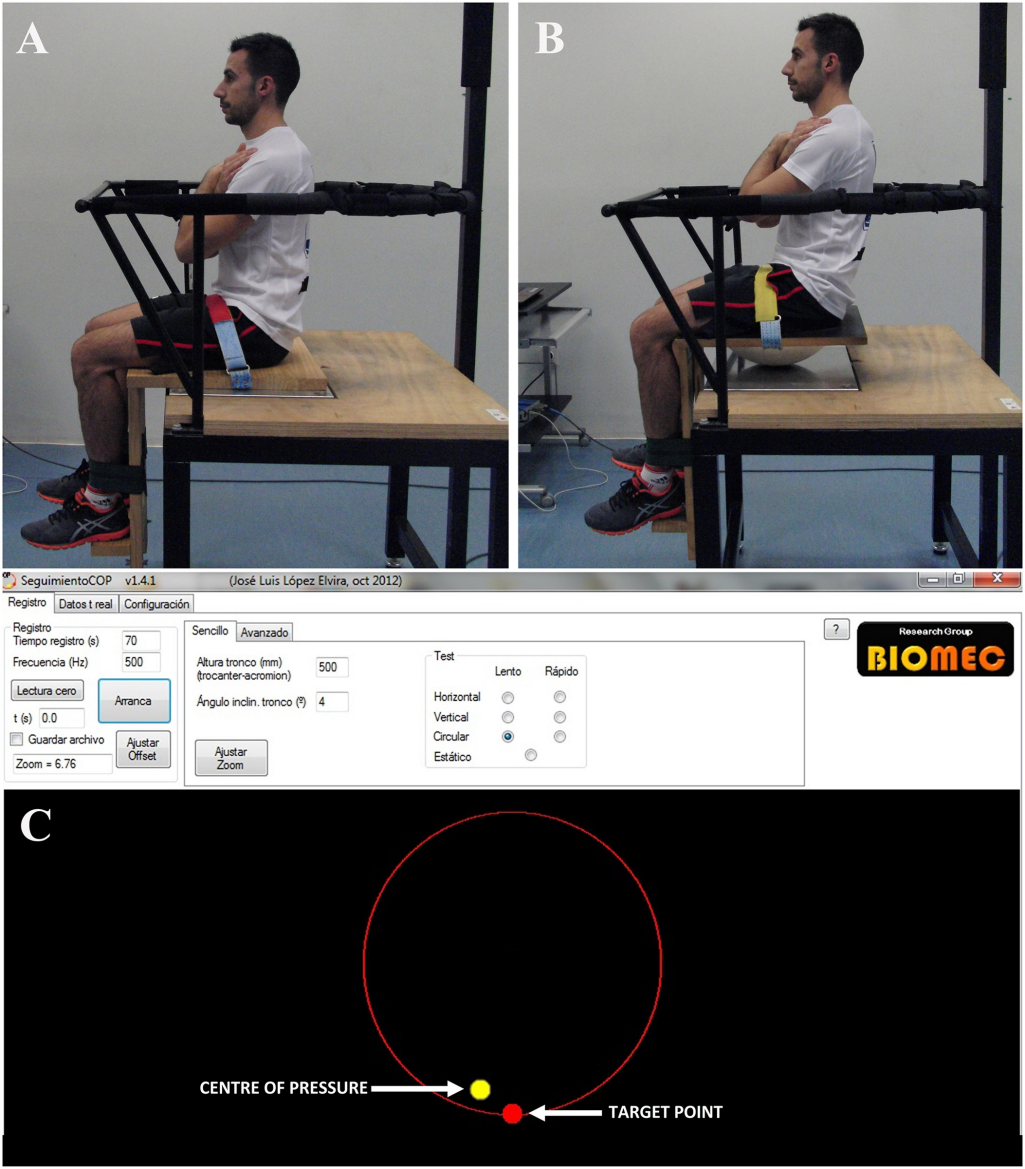
Participant performing a sitting task: 2A) on the stable seat; 2B) on the unstable seat; 2C) Projection providing visual feedback of participants’ centre of pressure and a target point moving across a circular path. The red path is shown in this picture to clarify the trajectory, but it was not presented to the subject during the trial.

2) *Stable and unstable sitting protocol* to assess participant’s ability to control trunk posture and motion while sitting. Participants performed different tasks while sitting on an unstable or a stable seat (Fig 2). The stable seat was a wooden structure with leg and foot supports. The foot support was adjusted to each participant (90° knee flexion) and the participant’s legs were strapped to the seat to prevent lower limb motions. The balance seat was the same structure with a polyester resin hemisphere attached to the bottom (hemisphere radius: 35 cm; height of the seat relative to the lowest point on the hemisphere: 12 cm). The seats were placed on a force plate (Kistler, Switzerland, Model 9286AA) located at 0.9 m height above the ground on a rigid, stable and flat surface. The force plate was sampled at 1000 samples/s. Feedback of the centre of pressure (CoP) displacement was provided to the participants in real time (Fig 2). In addition, a target point was presented to participants in several trials, to assess the subject’s ability to adjust his CoP position to the target location. During this protocol, participants performed 2 static and 3 dynamic trials on both seats. One of the static trials was performed without visual feedback, in which participants were asked to sit still in their preferred seated position; and the other trial was performed with visual feedback, in which participants were instructed to align their CoP position with the target point located in the centre of the screen. During the dynamic trials, participants were asked to track the target, which moved over three possible trajectories (anterior-posterior, medial-lateral and circular). During the dynamic conditions, the amplitude of target point displacement corresponded to a HAT centre of mass inclination angle of 4°. The target point took 20 seconds to complete a cycle (0.05 Hz). The duration of each trial was 70 seconds and the rest period between trials was 1 minute. Participants performed each trial with arms crossed over the chest. In the unstable conditions, all participants were able to maintain the sitting position without grasping a support rail. The full protocol was performed twice and 2 minutes of practice were given to participants before recording.

**Fig 3:**
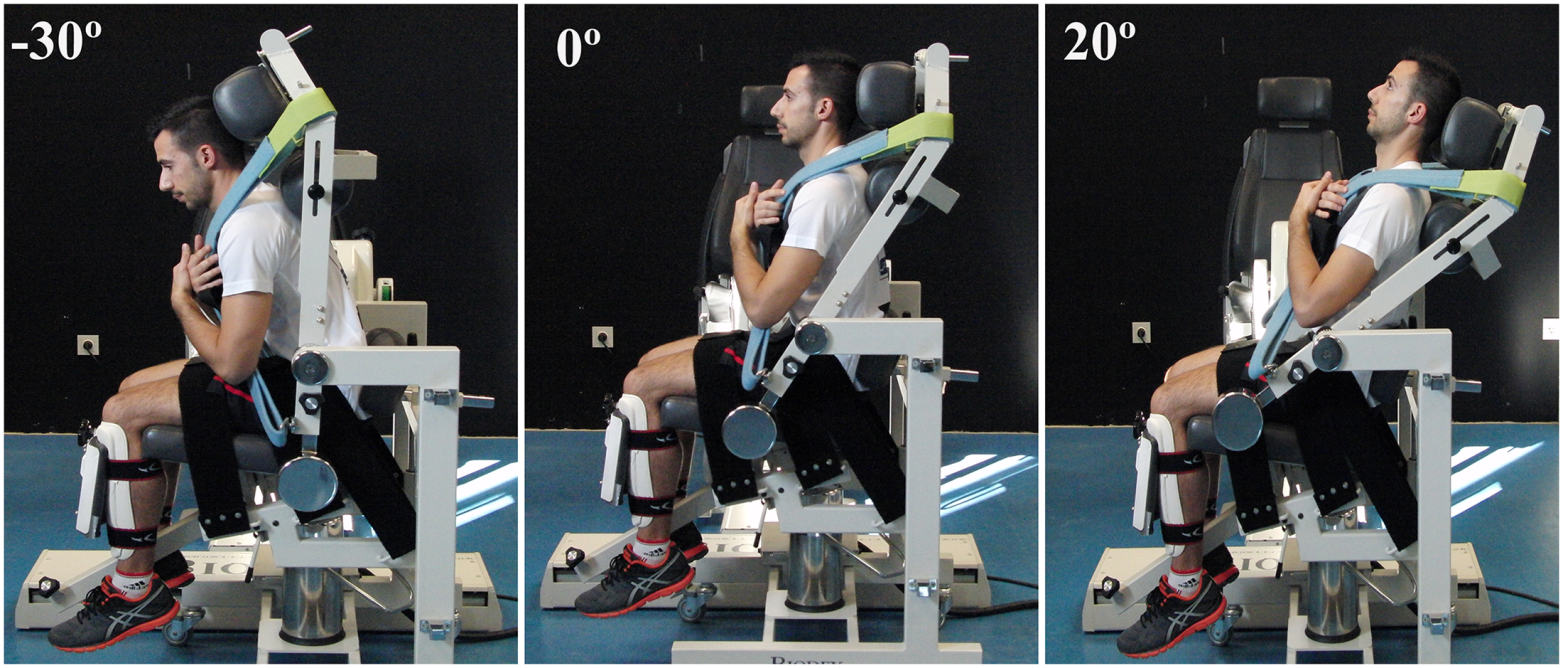
Participant performing maximum flexion-extension efforts in the isokinetic dynamometer throughout a 50° range of trunk motion: -30°) maximum trunk flexion; 0°) anatomical reference position; +20°) maximum trunk extension.

After the stability measures, a *trunk isokinetic protocol* was carried out in a Biodex^®^ isokinetic dynamometer (Model 2000, Multi-joint System 4 Pro, Biodex Corporation, Shirley, NY, USA) to assess trunk strength and endurance. Participants were seated in the *dual position back extension/flexion attachment* of the Biode^®^ isokinetic dynamometer (Fig 3). The participant’s trunk was placed upright with the hips and knees flexed at 90°, the thighs parallel to the floor and the dynamometer axis of rotation aligned with the imaginary line joining the anterior superior iliac spines. This was considered the anatomical reference position [16]. In order to fix the participant to the dynamometer attachment and ensure the protocol’s reliability, adjustable pads were placed behind the head, the sacrum and the upper-trunk and on the anterior surface of the tibia; in addition, the upper-trunk, the thighs and the pelvis were fixed with Velcro straps. The isokinetic test consisted of four sets of 15 consecutive maximal concentric trunk flexion and extension efforts at 120°/s. This flexion-extension speed was chosen because it is considered to be safe [17] and reliable for measuring mechanical work [18]. In each set, the motion started in the flexion direction and 1 minute rest was given between sets. Participants were instructed to keep their hands and arms crossed over the chest and were strongly encouraged throughout the test. The range of trunk motion was limited to 50° (Fig 3), i.e., from 30° of trunk flexion (-30°) to 20° of trunk extension (+20°) relative to the anatomical reference position (0°). According to Grabiner et al. (1990), ranges of trunk motion no larger than 50° isolate the lumbar motion, avoiding hip flexion-extension. In addition, the location of the dynamometer axis of rotation at the anterior superior iliac spine level, and the use of the pad behind the sacrum and the strap on the pelvis, minimized hip motion during the test.

Prior to testing, participants performed a warm-up which consisted of 5 minute cycling at 75 revolutions per minute on a stationary bicycle (Bike Excite Med TECHNOGYM), 2 sets of 15 crunches and 2 sets of 15 back extensions on a Roman chair. A 30 seconds rest was given between sets. Each measurement session lasted approximately 2 hours.

To characterize the response to sudden load moments applied to the torso, maximum trunk angular displacement (*θ*_*max*_), stiffness (*K)* and damping (*β*) were calculated according to Cholewicki et al. [19]. To estimate *K* and *β*, the trunk was represented as a second-order system with viscoelastic properties, oscillating freely after sudden perturbation [19]. In our data, *K* and *β* parameters had lowest error and highest reliability when 22 data points were analyzed (110 milliseconds). Therefore, taking into account that voluntary responses do not usually occur in the first 120–150 milliseconds [20] after perturbation, *K* and *β* obtained from the above method represent an effective stiffness and damping combining intrinsic muscle properties and reflex responses [19]. *θ*_*max*_, *K* and *β* were averaged over the best trials with the lowest *θ*_*max*_ values for each direction.

To quantify the trunk control during the sitting trials, we used the mean radial error (MRE). MRE was calculated as the average of vector distance magnitude (mm) of the CoP from the target point or from the participant’s own mean CoP position [21] for trials with and without visual feedback, respectively. The best of two trials performed for each condition (lower MRE) was used for subsequent statistical analyses.

To assess strength, peak torque (PT) was determined for each series and then the three highest PT were averaged for both, flexion and extension directions. In addition, based on Mayer et al. [22], the ratio of the highest amount of work recorded in any of the test repetitions and the mean work performed in the last three repetitions of the test (final fatigue ratio) was used to assess trunk flexion and extension endurance.

Descriptive statistics (mean and standard deviation) were calculated for each of the variables. Data normality was examined using the Kolmogorov-Smirnov statistic. One-way independent-measures analyses of covariance (ANCOVAs) were performed to investigate the differences between groups for all variables. Participants’ body mass was used as covariate (where it was applicable) to ensure that differences between groups were not influenced by anthropometry. ANOVAs were performed when participants′ body mass did not show a significant effect as covariate. Partial eta squared (${ƞ}_{p}^{2}$) was calculated as a measure of effect size. The Pearson correlation coefficient (r) was used to analyze the relationship between trunk strength, endurance and stability variables. All analyses were performed using the SPSS package (version 18, SPSS Inc., Chicago, IL, USA) with a significance level chosen at p<0.05.

## Results

The groups were not significantly different with respect to age, years of judo practice, height, mass and trunk moment of inertia. Regarding sudden loading parameters (Table 1), international judokas showed lower *θ*_*max*_ than national level athletes in the anterior direction. In addition, international level judokas tended to show higher *K* and lower *β* against anterior loading, but these differences were only close to significant (*K*: F = 4.194, p = 0.052; *β*: F = 3.906, p = 0.060). As can be seen in Table 1, no between-group differences in trunk movement control were found in stable and unstable sitting conditions. On the other hand, international level judokas showed higher PT during extension exertions than national level judokas (Table 1). However, no significant differences between groups in flexion strength variables were observed (Table 1). Finally, concerning trunk endurance, no significant differences between groups were found in final fatigue ratio during flexion and extension exertions (Table 1).

**Table 1:** Differences between national and international level judokas for trunk stability parameters obtained during sudden loading and sitting protocols and trunk isokinetic strength and endurance.

Protocols	Variables		National (n = 14)	International (n = 11)	F	*p*	${ƞ}_{p}^{2}$
***Sudden loading protocol***	***θ*_*max*_**	Anterior*	0.0981 ± 0.0184	0.0850 ± 0.0115	4.921	.037	.183
		**Lateral**	0.0685 ± 0.0154	0.0642 ± 0.015	.482	.494	.021
		**Posterior**	0.1990 ± 0.0224	0.1994 ± 0.0152	.001	.973	.000
	***K***	**Anterior**	1364.9 ± 342.9	1783.6 ± 662.8	4.194	.052	.154
		**Lateral**	1092.5 ± 343.8	1189.6 ± 379.8	.448	.510	.019
		**Posterior**	584.2 ± 104.2	587.7 ± 155.6	.005	.946	.000
	***β***	**Anterior**	334.7 ± 87.9	448.5 ± 192.0	3.906	.060	.145
		**Lateral**	687.5 ± 336.7	869.5 ± 336.7	2.576	.122	.101
		**Posterior**	100.9 ± 32.1	77.3 ± 38.2	2.848	.105	.110
***Sitting protocol***	**MRE**	**SSNF**	0.99 ± 0.36	0.98 ± 0.39	.007	.936	.000
		**SSWF**	0.76 ± 0.48	0.74 ± 0.23	.023	.882	.001
		**SSML**	2.38 ± 0.78	2.31 ± 0.41	.073	.789	.003
		**SSAP**	2.17 ± 0.44	2.13 ± 0.39	.052	.822	.002
		**SSCD**	3.42 ± 1.49	3.21 ± 0.68	.176	.679	.008
		**USNF**	5.68 ± 1.85	5.65 ± 1.83	.001	.970	.000
		**USWF**	4.87 ± 1.21	5.11 ± 1.56	.185	.671	.008
		**USML**	7.59 ± 2.19	7.01 ± 1.98	.466	.501	.020
		**USAP**	6.89 ± 1.25	7.11 ± 1.82	.123	.729	.005
		**USCD**	8.48 ± 1.61	8.81 ± 3.64	.096	.759	.004
***Isokinetic protocol***	**PT**	Extension*	399.1 ± 59.6	460.4 ± 62.9	9.372	.006	.299
		Flexion*	228.2 ± 25.0	212.1 ± 28.6	3.844	.063	.149
	**FFR**	**Extension**	76.6 ± 10.6	70.8 ± 11.6	1.682	.208	.068
		**Flexion**	62.3 ± 8.4	65.6 ± 9.2	.850	.366	.036

Independent measures ANOVA with between-subject factor with 2 levels (national and international).

* Independent measures ANCOVAs were performed using participant’s body mass as covariate.

PT = Peak torque (N*m); FFR = Final fatigue ratio (%).

*θ*_*max*_ (rad) = Trunk angular displacement; *K* (N*m/rad) = Trunk stiffness coefficient; *β* (N*m*s/rad) = Trunk damping coefficient.

MRE = Mean radial error (mm). Trunk sitting conditions: stable sitting without feedback (SSNF); stable sitting with feedback (SSWF); stable sitting while performing medial-lateral displacements with feedback (SSML); stable sitting while performing anterior-posterior displacements with feedback (SSAP); stable sitting while performing circular displacements with feedback (SSCD); unstable sitting without feedback (USNF); unstable sitting with feedback (USWF); unstable sitting while performing medial-lateral displacements with feedback (USML); unstable sitting while performing anterior-posterior displacements with feedback (USAP); unstable sitting while performing circular displacements with feedback (USCD).

Correlational analysis (Table 2) showed few significant correlations between strength, endurance and stability parameters, all of them below 0.512 and not always in the same direction.

**Table 2:** Correlations of trunk strength and endurance with trunk stability parameters obtained during sudden loading and sitting protocols.

Protocols	Variables		Strength (PT)		Endurance (FFR)	
			Extension	Flexion	Extension	Flexion
			*r* (*p*-value)	*r* (*p*-value)	*r* (*p*-value)	*r* (*p-*value)
***Sudden loading protocol***	***θ***_***max***_	**Anterior**	**-.419 (.037)**	-.052 (.807)	.259 (.210)	-.207 (.320)
		**Lateral**	-.058 (.784)	-.011 (.958)	.090 (.669)	-.006 (.979)
		**Posterior**	**-.466 (.019)**	-.281 (.173)	.289 (.160)	-.153 (.466)
	***K***	**Anterior**	.266 (.198)	-.037 (.862)	-.003(.989)	.363 (.075)
		**Lateral**	.269 (.193)	**.511 (.009)**	-.298 (.148)	.094 (.654)
		**Posterior**	.385 (.057)	.221 (.289)	**-.417 (.038)**	.218 (.296)
	***β***	**Anterior**	.264 (.202)	-.053 (.800)	**-.431 (.032)**	.341 (.096)
		**Lateral**	.096 (.649)	-.312 (.129)	.246 (.236)	-.326 (.111)
		**Posterior**	-.078 (.711)	.274 (.185)	-.380 (.061)	.204 (.328)
***e Sitting protocol***	**MRE**	**SSNF**	.018 (.931)	.083 (.693)	-.086 (.684)	.217 (.297)
		**SSWF**	-.163 (.435)	-.091(.664)	-.115 (.584)	.095 (.652)
		**SSML**	.028 (.893)	.077 (.713)	-.071 (.737)	.236 (.257)
		**SSAP**	-.254 (.220)	-.132 (.530)	-.295 (.153)	**.451 (.024)**
		**SSCD**	.014 (.946)	.103 (.625)	-.188 (.369)	.068 (.745)
		**USNF**	.137 (.513)	.069 (.742)	.025 (.904)	-.049 (.816)
		**USWF**	.086 (.684)	-.080 (.704)	.173 (.409)	-.153 (.467)
		**USML**	-.140 (.503)	-.004 (.986)	.116 (.581)	-.140 (.506)
		**USAP**	.154 (.462)	-.055 (.795)	-.080 (.704)	-.098 (.641)
		**USCD**	.157 (.454)	.033 (.874)	-.203 (.330)	-.052 (.807)

PT (N*m) = Peak torque; FFR (%) = Final fatigue ratio.

*θ*_*max*_ (rad) = Trunk angular displacement; *K* (N*m/rad) = Trunk stiffness coefficient; *β* (N*m*s/rad) = Trunk damping coefficient.

MRE = Mean radial error (mm). Trunk sitting conditions: stable sitting without feedback (SSNF); stable sitting with feedback (SSWF); stable sitting while performing medial-lateral displacements with feedback (SSML); stable sitting while performing anterior-posterior displacements with feedback (SSAP); stable sitting while performing circular displacements with feedback (SSCD); unstable sitting without feedback (USNF); unstable sitting with feedback (USWF); unstable sitting while performing medial-lateral displacements with feedback (USML); unstable sitting while performing anterior-posterior displacements with feedback (USAP); unstable sitting while performing circular displacements with feedback (USCD).

## Discussion

Although it has been suggested that core muscle fitness is a key factor in high-level judo performance [4, 5] there is limited empirical evidence to support this. Therefore, the objective of the present study was to assess core strength, endurance and stability differences between high-level and medium-level judokas competing at international and national levels respectively. The major finding was that international level judokas showed higher isokinetic extension strength and better responses (lower trunk angular displacement) after sudden trunk loading in anterior direction than national level judokas.

Our results support the notion that core strength is a determinant of high-level judo performance. Specifically, international level judokas showed higher trunk extensor peak torque than national ones (Table 1). These results do not support previous findings by Iwai et al., in which no differences were found in isokinetic trunk extension and flexion strength between international (Olympic) and national level Japanese judokas. However, it is noteworthy that in our study, international level judokas showed higher trunk extensor isokinetic strength (average of PT relative to de body mass at 120°/s concentric contractions: 6.2 N*m/kg) than in the study by Iwai et al. (average of PT relative to the body mass at 120°/s concentric contractions: 5.5 N*m/kg), which may be the reason for the differences found between studies. The importance of the trunk extensors would be in line with previous statements that antigravity muscles are most intensively used during most throws in judo [23]. Specifically, trunk extensors play a main role in breaking the opponent’s balance during forward throw techniques in which judokas must unbalance their adversaries, moving the opponent’s centre of mass in forward and upward direction [24]. High trunk extensor strength could also be associated with the necessity of maintaining upright stance in the guard position (while coping with the continuous pulling perturbations by the adversary) to avoid both, falling and referee penalties for an excessively flexed defensive posture. It is important to highlight that during high-level competition, penalties can be a key factor in victories; for example, during the Olympic Games in Atlanta, the referees’ sanctions were the second decisive factor in wins [25].

Concerning core stability, international level judokas showed lower trunk angular displacement and tended to show greater trunk stiffness and damping after anterior loading than national level judokas, but no differences were found after lateral and posterior loading (Table 1). The better responses in international level judokas after anterior loading could be explained by the same ideas presented above about the maintenance of upright stance against the opponent’s pulling disturbances. Taking into account that the time window after the perturbations used in our analysis was 110 milliseconds, our results could be related with the judokas effort to keep the balance during the balance breaking phase of forward throw techniques, which occurs within the first 150 milliseconds of these throws [24]. International level judokas’ smaller trunk displacement after anterior loading was associated with a trend towards higher trunk stiffness and damping. It is important to remark that trunk stiffness and damping may not only reflect passive tissue properties, but also active reflexive control of posture. In this sense, higher damping in international judokas could be related to a higher ability to dissipate perturbation forces, which are usually applied by the adversary with the intention of causing a reaction in the opposite direction to disturb the opponent’s balance [26]. However, the difference in trunk damping and stiffness between groups did not reach significance, so further research is necessary to confirm this hypothesis.

Considering strength and stability results together, trunk extensor strength and stability against trunk anterior loading could be important factors to avoid the opponents’ pulling perturbations. However, the few significant correlations found between these variables (Table 2) suggests that both are different features of trunk muscle function, which must be trained specifically in competitive judo.

Regarding the balancing test while sitting, it could be considered a non-specific test to assess core stability in judokas, given the posture in which the test takes place and given the unstable surface used in part of the protocol. The lack of differences between groups (Table 1) supports previous findings by Barbado et al. [27] in which competitive judokas obtained better results than competitive kayakers and recreational athletes only in a sudden loading protocol, but they did not obtain better results in an unstable sitting test, in which kayakers showed the best scores. These and our results indicated that core stability tests need to be designed to reflect sport-specific demands.

Concerning trunk extension and flexion isokinetic endurance, no differences were observed between groups (Table 1). Although previous studies have emphasised the importance of anaerobic and trunk endurance for performance in judo, [10–12, 28, 29] our results seem to indicate that trunk local muscle fatigue is not a limiting factor in high-level judo competition. However, a previous study on this isokinetic protocol showed that absolute and relative reliability of the final fatigue ratio was moderate [30], and therefore the comparison between groups for this variable should be interpreted carefully.

Regarding the correlational analysis, the few and small significant correlations found (Table 2) suggest that trunk strength and endurance are not limiting factor for trunk stability in this population. However, in our study trunk responses against sudden perturbations were assessed against a single submaximal load magnitude and in an unfatigued condition. Future studies should assess the relationship between these variables over a wider range of perturbations and in fatigued and non-fatigued conditions.

The present study presents some limitations which could bias the results presented above and their interpretations. Although the sample size was not small according to the judokas’ high sport level, more studies are necessary to generalize our findings. In addition, aspects of trunk muscle performance that did not differentiate between groups in the current study may still be important for judo performance and would for example be different between recreational judokas and competitive judokas. In this sense, to obtain a better understanding of trunk muscle performance in competitive judokas, future studies should assess the relationship between trunk parameters and specific judo skills rather than overall sport performance criteria (i.e., ranking, level of competition, etc.) which can be strongly influenced by many variables. Moreover, as several physical capabilities are needed to be successful in judo competition, futures studies on trunk muscle function should include the assessment of most of these physical capabilities (e.g., upper and lower body strength, speed, anaerobic power, etc.) and explore their interactions and their influence on judo performance. Finally, further research is necessary to evaluate the judokas’ voluntary responses against expected and unexpected trunk loading, as considering the sport demands it could be relevant to judo performance.

## Conclusions

The present study examined the differences in core strength, endurance and stability between international and national level of competitive judokas. International level judokas presented higher trunk extensor strength and better responses after sudden trunk loading in anterior direction than national level judokas, suggesting that these variables should be considered key components of training programs for elite judo athletes. However, although certain levels of trunk muscle endurance and trunk balance control are undoubtedly necessary to train and compete in judo, they do not seem to represent a limiting factor in elite judo performance, as no differences were found in these variables between international and national level judokas. Finally, the lack of significant correlations between trunk strength, endurance and stability parameters suggests that trunk stability performance is not limited by trunk strength and/or endurance in the testing conditions.

## Supporting Information

S1 Data Raw data of all variables analysed in this study. (XLSX)
