# Correction: Trunk Stability, Trunk Strength and Sport Performance Level in Judo

**DOI:** 10.1371/journal.pone.0162962

**Published:** 2016-09-09

**Authors:** David Barbado, Alejandro Lopez-Valenciano, Casto Juan-Recio, Carlos Montero-Carretero, Jaap H. van Dieën, Francisco J. Vera-Garcia

The third sentence of the sixth paragraph in the Discussion section should have cited reference 22 instead of 30.

The correct sentence should read: However, a previous study on this isokinetic protocol showed that absolute and relative reliability of the final fatigue ratio was moderate [22], and therefore the comparison between groups for this variable should be interpreted carefully.

Reference 30 is cited in error in the published article and should be removed. Reference 30 is: Juan-Recio C, Lopez-Plaza D, Barbado D, Garcia-Vaquero MP, Vera-Garcia FJ. Reliability and relationship of an isokinetic and two field tests to assess trunk muscle performance. Journal of strength and conditioning research. 2016; (In press).
